# Field-Recovered Polystyrene Exposure Alters Developmental Endocrine Signaling and Cholinergic Function in the Plastivorous Insect *Zophobas morio*

**DOI:** 10.3390/ijms27198794

**Published:** 2026-09-30

**Authors:** Paolo Cocci, Mario Marconi, Gilberto Mosconi, Francesco Alessandro Palermo

**Affiliations:** School of Biosciences and Veterinary Medicine, University of Camerino, Via Gentile III Da Varano, I-62032 Camerino, MC, Italy; mario.marconi@unicam.it (M.M.); gilberto.mosconi@unicam.it (G.M.); francesco.palermo@unicam.it (F.A.P.)

**Keywords:** endocrine disruptors, *Zophobas morio*, marine litter, gene biomarkers

## Abstract

The use of insect larvae for plastic waste biodegradation has attracted increasing attention as a potential strategy to mitigate environmental plastic pollution. However, the toxicological consequences of ingesting plastics recovered from the environment, particularly those acting as vectors for sorbed contaminants, remain poorly understood. In this study, we investigated the endocrine and neurotoxic effects of field-recovered (environmental) polystyrene (ePS) in the plastivorous insect *Zophobas morio*. Larvae were fed virgin PS (vPS) or PS collected from a marine coastal area known to be impacted by anthropogenic activities. Chemical analyses revealed that ePS was characterized by a mixture of organochlorine and organophosphorus pesticides, with a total complex residue concentration of 23.7 ± 5.5 µg g^−1^, whereas only trace levels of hexachlorocyclopentadiene (HCCPD) were detected in vPS (2.55 ± 0.65 µg g^−1^). All compounds detected in ePS were also detected in larvae from the ePS-fed group, indicating effective trophic transfer from plastic to the organism. Despite the absence of mortality, PS-fed larvae exhibited reduced growth compared to controls. At the molecular level, ingestion of ePS caused a significant downregulation of key genes involved in the ecdysteroid signaling pathway (ecdysone receptor, EcR; ultraspiracle, USP; and early response genes E74 and E75), coupled with an upregulation of juvenile hormone-responsive genes (methoprene-tolerant, Met; Krüppel homolog 1, Kr-h1), suggesting endocrine imbalance potentially affecting developmental progression. In parallel, acetylcholinesterase (AChE) enzymatic activity was significantly inhibited, particularly in larvae fed ePS, despite no changes in AChE or acetylcholine receptor transcript levels, indicating functional neurotoxicity. This study highlights the importance of considering plastic-associated contaminant toxicity when evaluating insect-based plastic biodegradation strategies.

## 1. Introduction

The potential of using insect larvae, particularly those of *Tenebrio molitor* (yellow mealworm) and *Zophobas atratus/morio* (superworms), for the biodegradation of plastic waste has been largely reported in the recent literature [1,2,3]. Several studies highlight the ability of these larvae to consume and degrade various types of plastics (e.g., expanded polystyrene (EPS) and low-density polyethylene (LDPE)), thus supporting the ecological significance of utilizing insects in waste management strategies [4,5]. This approach is particularly relevant within circular economy frameworks, as it could contribute not only to mitigating plastic pollution but also to promoting more sustainable food systems through the use of insects as a source of protein and other valuable nutrients [6]. Nowadays, few studies have investigated the potential toxicological implications of plastic waste biodegradation on insect health, which instead is crucial for producing harmless insect biomass for possible application in feed and food production. In this regard, plastic toxicity refers to the harmful effects that plastics and their additives can have on plastivores. This toxicity arises from both the physical presence of plastic materials in the gut and the chemical substances associated with them. Specifically, plastic biofragmentation results in the production of micro- and nano-plastic pieces that can fill the gastrointestinal tract of insects, leading to impaired essential nutrient utilization, altered microbiota, cell damage and oxidative stress [7]. Plastics often contain additives (e.g., phthalates and flame retardants) that can leach into the organisms’ gastrointestinal tracts, leading to toxic effects such as endocrine disruption [8,9]. In addition, it has been recently demonstrated that plastic waste can absorb harmful environmental pollutants that can be released upon ingestion by insects, producing further toxicity [10]. The interaction between plastics and environmental pollutants can enhance the uptake of these harmful compounds. Plastic waste can adsorb a variety of environmental pollutants, including emerging contaminants and pesticides. For example, the co-application of polyvinyl chloride microplastics (MPs) and perfluoroalkyl substances resulted in a significantly higher pollutant uptake in earthworms (*Eisenia fetida*), thus affecting their reproductive capacity [11]. Similarly, the ingestion of MPs associated with sorbed pesticides may enhance toxic responses compared with exposure to either MPs or pesticides alone. In terrestrial ecosystems, co-exposure to MPs and pesticides has been shown to induce oxidative stress and disrupt metabolic processes in earthworms. This includes elevated lipid peroxidation levels and changes in enzyme activities, indicating adverse effects on the health [12,13,14]. Studies have shown that simultaneous exposure to MPs and herbicides (i.e., atrazine) can lead to increased oxidative stress in *Eisenia fetida*, resulting in abnormal expression of genes considered sensitive biomarkers for environmental toxicology monitoring [15]. In three terrestrial species (*Enchytraeus crypticus*, *Folsomia candida*, and *Porcellionides pruinosus*), exposure to nanoplastics resulted in oxidative stress and led to redox imbalance and increased neurotoxicity by decreasing acetylcholinesterase (AChE) activity [16]. In this regard, we have recently demonstrated that feeding superworms with PS collected from a marine coastal area produced oxidative damage and lipid peroxidation [2]. The combination of MPs with pesticides showed an adverse effect on the growth of several earthworm species, including *Eisenia fetida*, *Aporrectodea caliginosa*, and *Lumbricus terrestris* [17]. Pesticides have also been shown to act as endocrine disruptors affecting various biological processes, including development through their effects on oxidative stress and the ecdysteroid signaling pathway. In insects, two biological systems particularly susceptible to environmental toxicants are the ecdysteroid hormone signaling pathway and the cholinergic neurotransmission system. The ecdysteroid signaling pathway plays a central role in regulating molting and metamorphosis and is regulated through a tightly controlled gene network. The ecdysone receptor (EcR) forms a heterodimer with ultraspiracle (USP) to bind specific hormone response elements and induce transcription of early response genes such as E74 and E75. These, in turn, activate downstream targets including methoprene-tolerant (Met) and Krüppel homolog 1 (Kr-h1), which mediate critical transitions during larval development. Exposure to endocrine-disrupting chemicals (EDCs), such as certain pesticides commonly associated with plastic debris, can dysregulate these transcriptional events, leading to developmental arrest, impaired molting, or reduced fecundity [15,18] The cholinergic system represents another conserved and sensitive target for environmental neurotoxins. Acetylcholine (ACh) is a principal neurotransmitter in both the central and peripheral nervous systems of insects. Its action is terminated by AChE, which hydrolyzes ACh into choline and acetate. Organophosphate and carbamate pesticides, frequently detected on weathered plastic, are potent inhibitors of AChE, leading to accumulation of ACh in the synaptic cleft, continuous stimulation of cholinergic receptors, and neurotoxic effects including tremors, muscle paralysis, and mortality. In addition to direct enzyme inhibition, exposure to such compounds may also affect gene expression levels of ACh, AChR, and other components of the cholinergic signaling cascade. In this study, we investigated the biological effects of dietary exposure to field-recovered (environmental) polystyrene (ePS) in *Zophobas morio* larvae, with particular attention to plastic-associated pesticide contaminants. Specifically, we aimed to: (1) characterize the pesticide profile associated with ePS collected from a marine coastal area and assess the transfer of detected compounds to exposed larvae; (2) evaluate larval survival and growth; and (3) investigate potential alterations in endocrine-related signaling by assessing genes involved in the ecdysteroid pathway (EcR, USP, E74 and E75) and juvenile hormone signaling (Met and Kr-h1), together with cholinergic endpoints, including AChE and AChR gene expression and AChE enzymatic activity.

## 2. Results

### 2.1. Profiles of the Pesticides Associated with Field-Recovered PS

The total concentration of organophosphorus (OP) and organochlorine (OC) pesticide residues in ePS was 23.7 ± 5.5 µg/g (Table 1). Among OC pesticides, we found that four of them (i.e., hexachlorocyclopentadiene, atrazine, γ-chlordane and methoxychlor) were present in the mean range of 1.85 to 8.65 µg/g with the highest concentration corresponding to HCCPD. Interestingly, this compound was also present in vPS at lower levels (i.e., 2.55 µg/g). In relation to the group of OP pesticides, we detected both MOCAP and RONNEL at mean concentrations of 4.26 and 3.69 µg/g, respectively. No organophosphorus pesticides were detected in vPS. All six compounds detected in ePS were also detected in larvae from the ePS-fed group (Table 1). Some pesticide concentrations in the whole body of larvae exposed to ePS ranged between 2.7 and 8.3 µg/g with a median value of 4.77 µg/g. The observed OC pesticide pattern was quite similar to that in the ingested ePS, with HCCPD as the dominating compound, followed by γ-chlordane, methoxychlor and atrazine. Regarding OP, MOCAP exhibited higher bioaccumulation levels compared to RONNEL, with mean concentrations of 1.09 µg/g and 0.78 µg/g, respectively. In the vPS fed group, the sole pesticide detected was HCCPD, with concentrations ranging from <loq to 0.33 µg/g (Table 1).

### 2.2. Effects of PS and Pesticides on Superworm Mortality and Body Size

**Figure 1:**
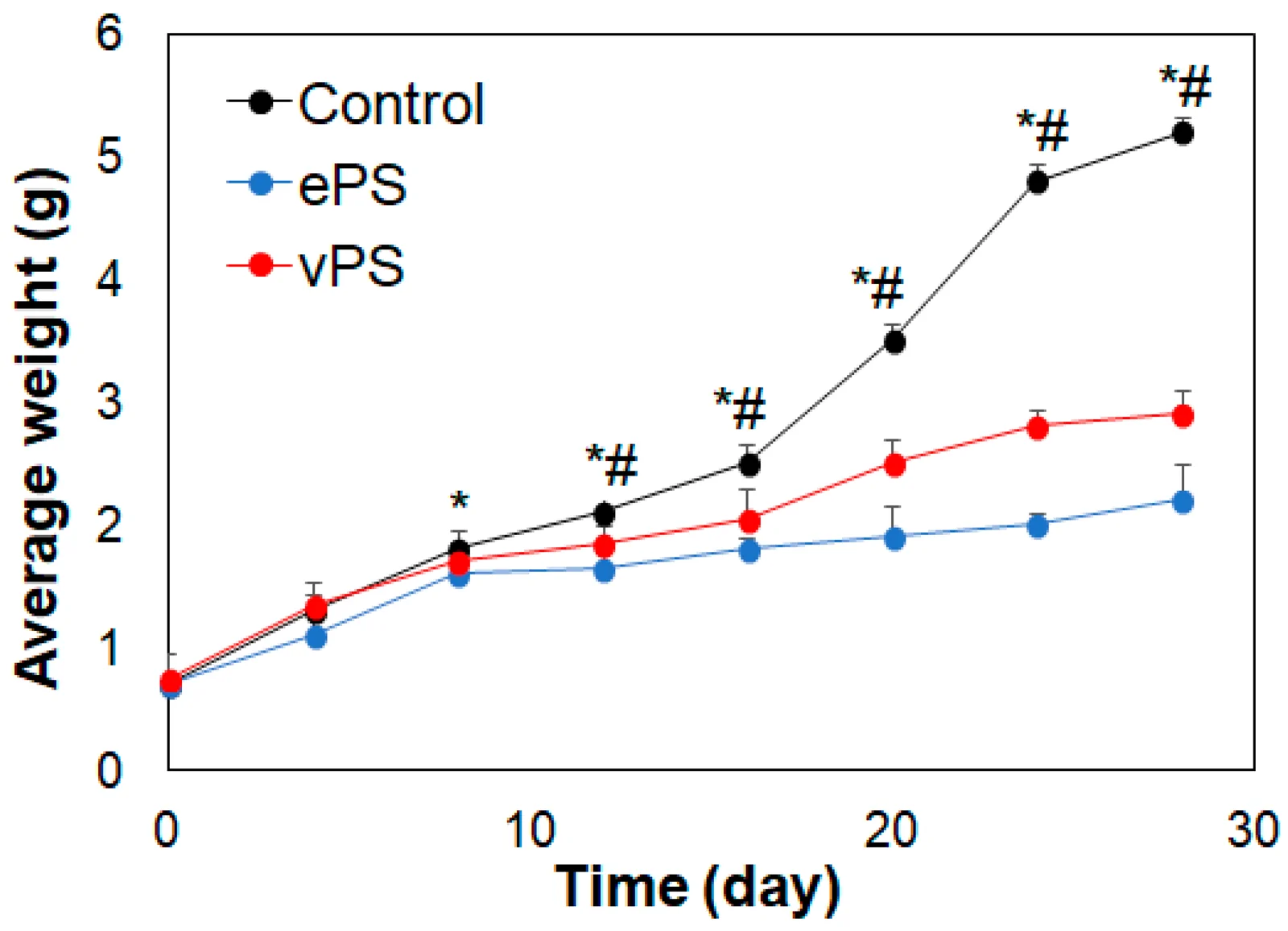
Average body weight (g) of *Zophobas morio* larvae exposed to virgin polystyrene (vPS) or field-recovered (environmental) polystyrene (ePS). Data are expressed as mean ± standard deviation. Individual measurements from the replicate rearing boxes were combined for graphical representation, while treatment comparisons were performed at the experimental-unit level. * *p* < 0.05 ePS vs. control; # *p* < 0.05 vPS vs. control.

No mortality was detected. The mass loss of PS was 35 ± 6%, and 55 ± 5% for the vPS-fed and ePS-fed, respectively. The growth rates of groups fed with PS were both significantly lower than those of the control group during the feeding trial. No statistically significant differences were detected between the groups fed with ePS and those fed with vPS (Figure 1).

### 2.3. mRNA Expression of Ecdysteroid Biosynthetic Pathways/Cholinergic System-Involved Genes and AChE Enzymatic Activity in Response to PS

**Figure 2:**
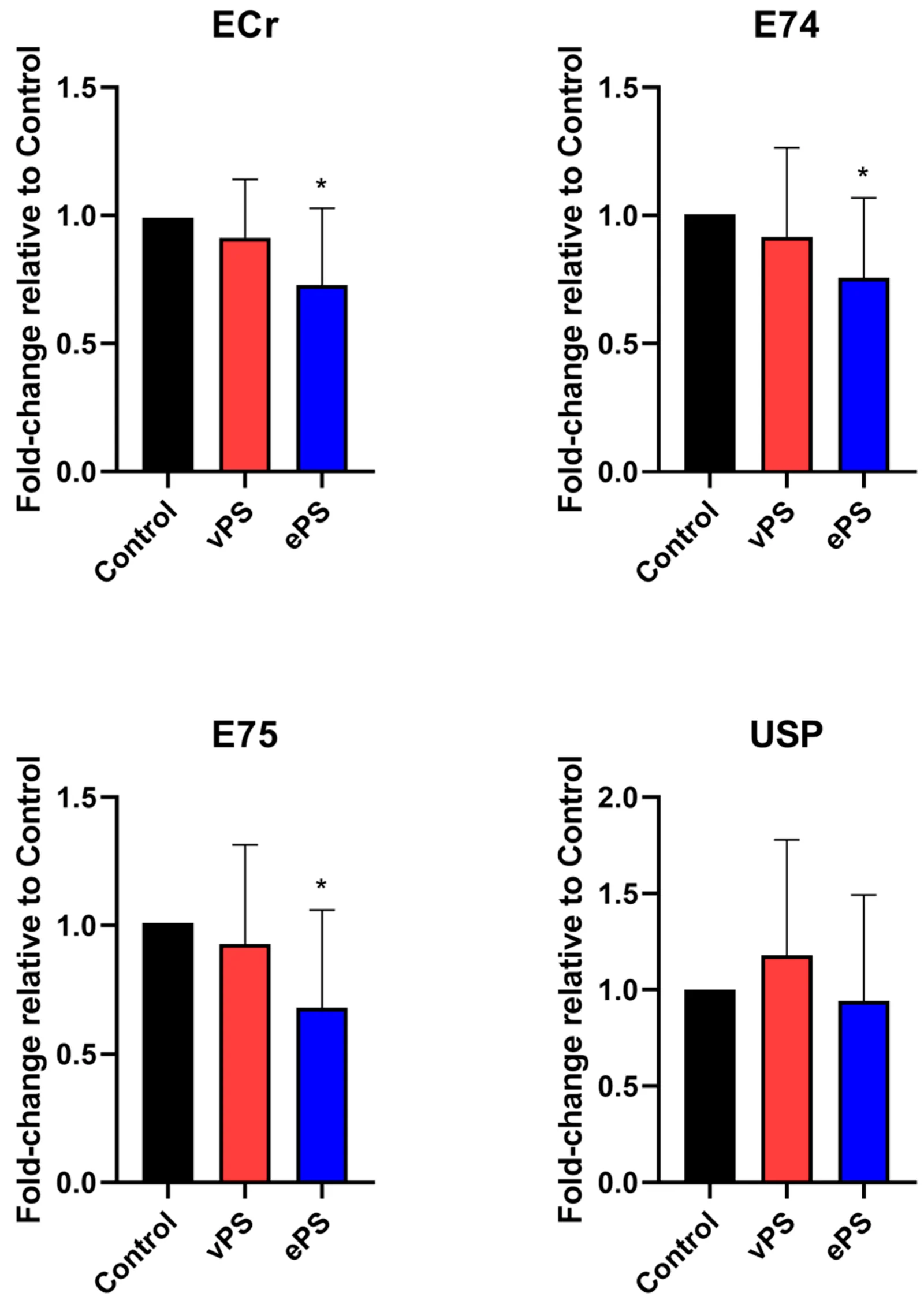
Fold change in the expression of genes involved in the ecdysteroid biosynthetic pathway in *Zophobas morio* larvae exposed to virgin polystyrene (vPS) or field-recovered (environmental) polystyrene (ePS). Gene expression levels were normalized to the control group. Individual measurements from the replicate rearing boxes were combined for graphical representation, while treatment comparisons were performed at the experimental-unit level. * *p* < 0.05 versus control.

To investigate the effects of PS on ecdysteroid and juvenile hormone (JH) signaling pathways, as well as the cholinergic system, mRNA expression levels of genes involved in 20-hydroxyecdysone and JH signaling (i.e., EcR, USP, E74, E75, Met, and Kr-h1) and genes encoding acetylcholinesterase (AChE) and acetylcholine receptor (AChR) were quantified in larvae fed with virgin vPS and ePS. In larvae exposed to ePS, a significant downregulation of most genes associated with the ecdysteroid signaling pathway was observed compared to the control group (*p* < 0.05) (Figure 2).

**Figure 3:**
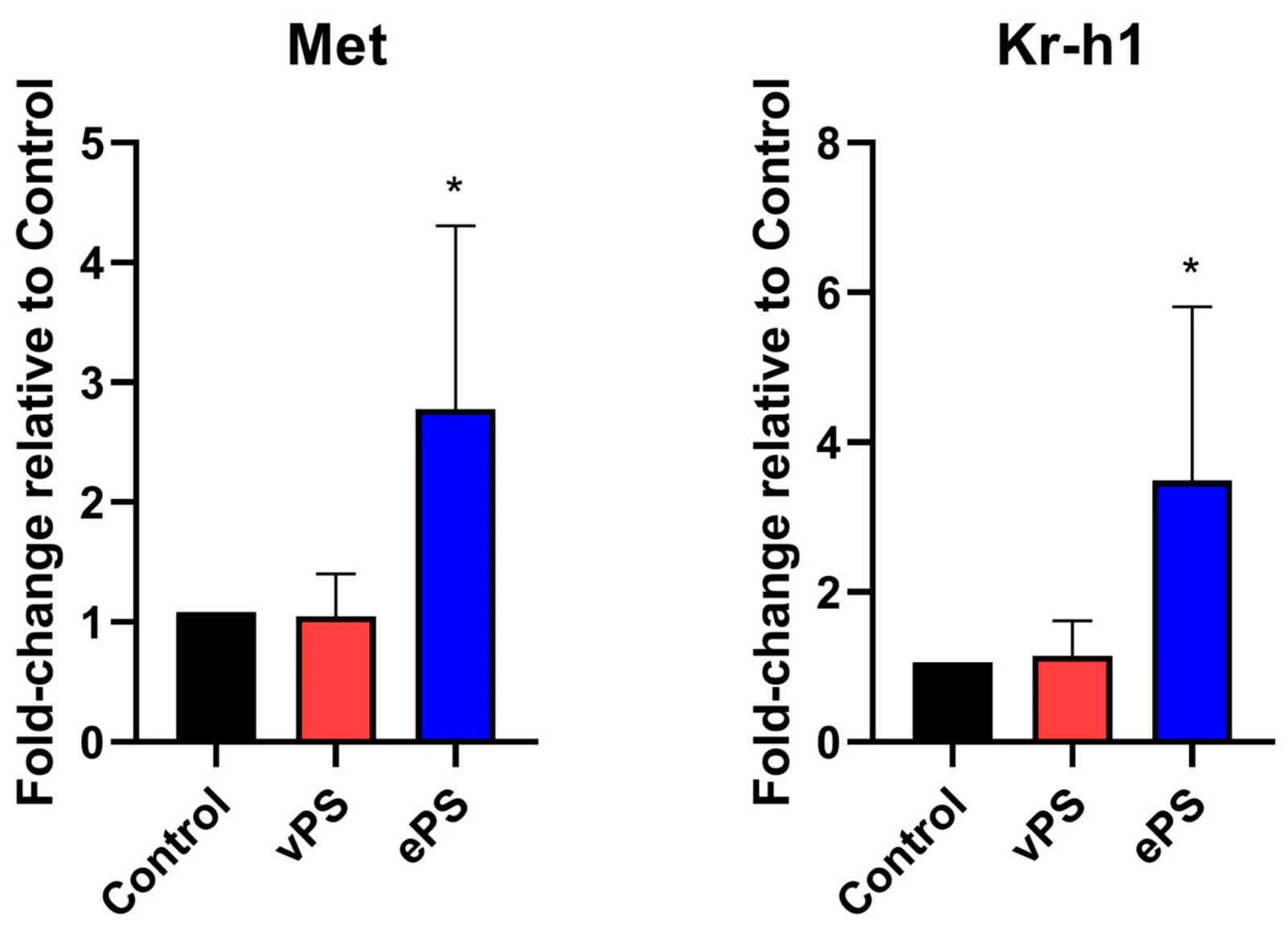
Fold change in the expression of juvenile hormone target genes (Met and Kr-h1) in *Zophobas morio* larvae exposed to virgin polystyrene (vPS) or field-recovered (environmental) polystyrene (ePS). Gene expression levels were normalized to the control group. Individual measurements from the replicate rearing boxes were combined for graphical representation, while treatment comparisons were performed at the experimental-unit level. * *p* < 0.05 versus control.

Conversely, ingestion of ePS resulted in upregulation of JH target genes (i.e., Met and Kr-h1) relative to both control and vPS groups, suggesting potential disruption of larval development and metamorphosis (Figure 3).

**Figure 4:**
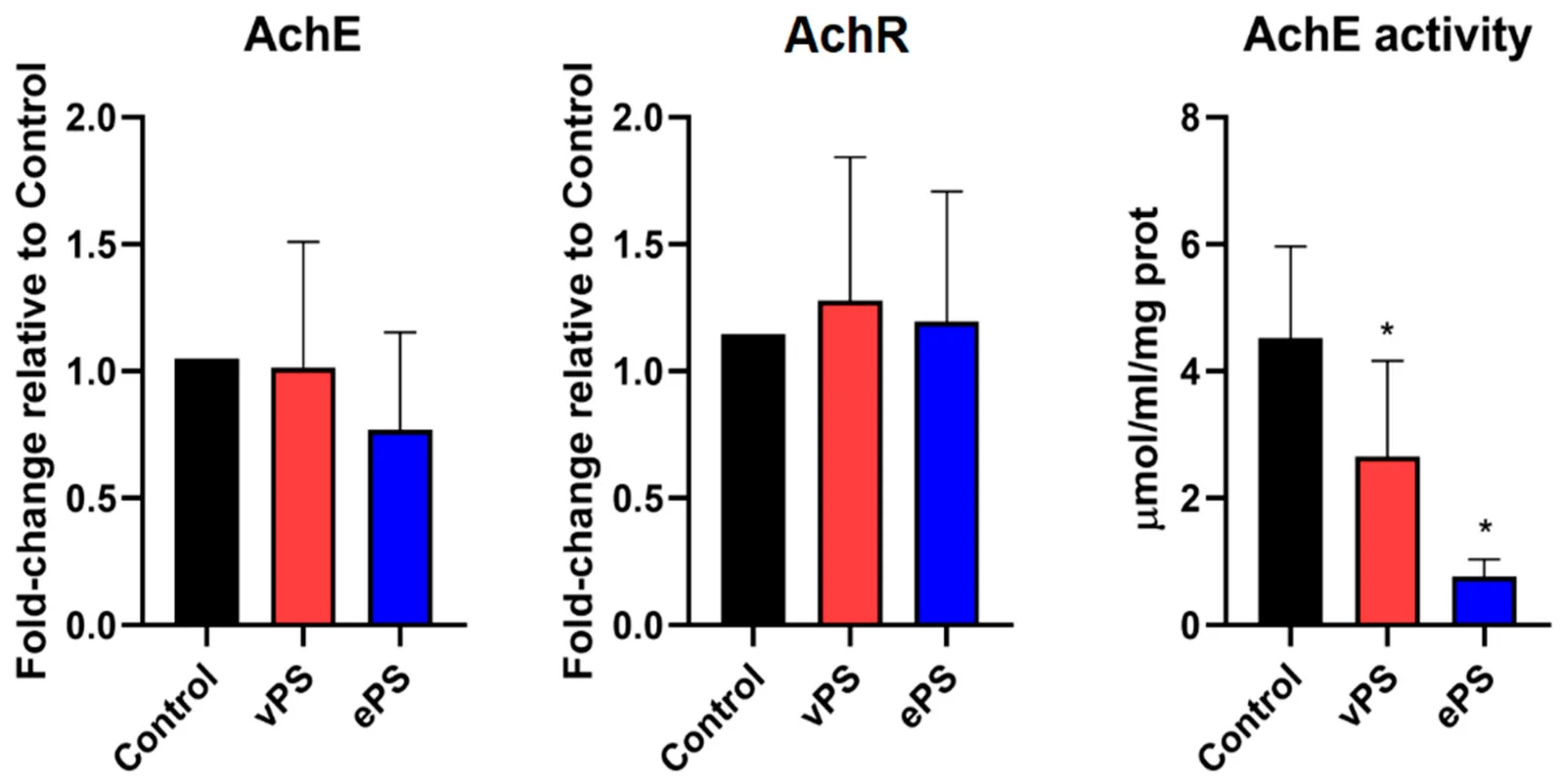
Gene expression of acetylcholinesterase (AChE) and the acetylcholine receptor (AChR), together with AChE enzymatic activity, in *Zophobas morio* larvae exposed to virgin polystyrene (vPS) or field-recovered (environmental) polystyrene (ePS). Individual measurements from the replicate rearing boxes were combined for graphical representation, while treatment comparisons were performed at the experimental-unit level. * *p* < 0.05 versus control.

Furthermore, PS ingestion correlated with a significant inhibition of AChE enzymatic activity, despite no observed changes in AChE mRNA expression or AChR gene levels, with the greatest inhibition detected in ePS-fed larvae (Figure 4).

## 3. Discussion

Evaluating the impact of field-recovered polystyrene on plastivorous insects is crucial for understanding the potential toxicological consequences of plastic waste biodegradation on insect health. This knowledge supports the development of strategies and interventions aimed at mitigating plastic pollution while promoting the production of non-toxic insect biomass, with potential applications in feed and food production. Accordingly, the present study offers scientific evidence on how PS waste, collected from marine environments, affects the physiological health of key insect species commonly employed in plastic biodegradation processes. It is well known that one of the most common sources of marine litter, especially in our study area, is PS derived from horticultural seedling production and marine aquaculture activities. These materials often degrade into smaller fragments due to environmental exposure and improper disposal, contributing significantly to coastal and oceanic plastic pollution [19,20]. At the same time, aging processes, such as ultraviolet radiation, enhance surface roughness and increase oxygen-containing functional groups, thereby strengthening the carrier capacity of microplastics. Hydroxyl and carboxyl functional groups on microplastic surfaces represent key binding sites for a wide range of persistent organic pollutants, including pesticides, PCBs, PAHs and flame retardants, acting as vectors for multiple contaminants [21].

An important preliminary observation of the present study is that the modified experimental setup, characterized by reduced humidity and stocking density, effectively minimized larval mortality throughout the exposure period. This improvement in rearing conditions provided a more stable experimental framework and allowed treatment-related differences in larval growth to be more clearly evaluated. The absence of mortality in the present study, unlike that observed previously in Cocci et al. [2], therefore enabled a more reliable assessment of growth responses across experimental groups. Moreover, we confirmed the previously reported greater consumption of ePS relative to vPS. This behavior may reflect physical and biological modifications acquired by ePS during environmental exposure, potentially enhancing its fragmentation and accessibility to larval feeding.

The pesticide profile detected in ePS demonstrates that field-recovered PS can carry a complex mixture of environmentally associated contaminants. The total pesticide load measured in ePS falls within the range reported for environmental microplastics collected in contaminated terrestrial and coastal systems, where sorption of hydrophobic pesticides on aged plastics is well documented [22]. The dominance of HCCPD in ePS, and its presence, although at lower concentration, in vPS, suggests that polystyrene may already contain legacy contaminants or that production-related impurities persist at low levels [23]. This aligns with previous findings showing that primary pellets often retain manufacturing-associated organochlorines, while secondary fragments accumulate pollutants from the environment (as reported for DDT metabolites, chlordane and other OCPs in coastal debris) [24]. The presence of OP pesticides (MOCAP and RONNEL) in ePS and their complete absence in vPS confirm that these compounds were acquired through environmental sorption. This is consistent with experimental evidence showing that aged plastics exhibit higher affinity for OPs and other relatively hydrophobic pesticides due to well-known processes such as increased surface oxidation, microporosity and functional group enrichment induced by UV and mechanical weathering [25].

All targeted pesticides were detected in larvae, with a whole-body concentration range consistent with MP-mediated delivery demonstrated in soil invertebrates and fish. Studies show that aged MPs enhance both the sorption and subsequent biological availability of pesticides, particularly when MPs undergo fragmentation and oxidation, increasing their capacity to carry hydrophobic contaminants into organisms [22,25]. The similarity between the OC profile in ePS and that in exposed larvae strongly supports the hypothesis that ePS serves as a direct vector for contaminant transfer upon ingestion. In marine environments, a comparable mirroring of pesticide profiles between microplastic debris and exposed organisms has been demonstrated, particularly for persistent and highly hydrophobic OCPs such as DDTs and chlordane derivatives [24]. Notably, OPs were detected in all larvae, with MOCAP showing greater bioaccumulation than RONNEL. This selective uptake reflects established patterns where MPs modify the internal exposure levels of pesticides depending on sorption affinity, desorption behavior in digestive fluids, and particle size [25]. Overall, the strong overlap between pesticide patterns in ePS and in exposed larvae confirms the direct trophic transfer of adsorbed pesticides via ingestion, and the ecological relevance of weathered PS as a carrier, consistent with observations from terrestrial and marine investigations in which MPs exhibit pollutant loads reflecting the environmental matrix from which they originate. On the other hand, data also demonstrate that the exclusive detection of HCCPD in larvae fed vPS is consistent with the findings that some OCPs (e.g., DDT metabolites, HCHs) may already be present in virgin pellets from production and transport processes, as previously observed in Canary Island plastics and industrial pellets [24]. This small but measurable baseline underscores that even “clean” PS may carry legacy contaminants, but the magnitude is orders of magnitude lower than that observed in ePS-fed larvae. Exposure to ePS induced a complex pattern of molecular alterations affecting endocrine signaling and cholinergic function in the larvae. A marked downregulation of key components of the ecdysteroid signaling pathway (i.e., EcR, USP, E74, and E75) was observed in ePS-fed individuals, indicating an altered transcriptional regulation of 20-hydroxyecdysone-mediated developmental signaling. Similar transcriptional repression of ecdysteroid-related genes has been documented in *Tigriopus japonicus* exposed to atrazine [26], where downregulation of EcR and USP was associated with altered molting and impaired metamorphosis. These findings collectively support the interpretation that ePS acts as carriers of chemical pollutants capable of disrupting the hormonal cascade that regulates larval development. Conversely, larvae exposed to ePS showed a marked upregulation of juvenile hormone-responsive genes (i.e., Met and Kr-h1). Since JH signaling antagonizes ecdysteroid action, the observed reciprocal modulation of these pathways suggests an endocrine imbalance favoring maintenance of the larval state [27]. The induction of Kr-h1, which is widely recognized as a molecular marker of JH activity, further points to a developmental delay mechanism analogous to that described in insects and crustaceans under exposure to endocrine-disrupting chemicals [28]. Although the expression of these endocrine-related genes is intrinsically linked to developmental progression, morphological assessment indicated a comparable larval developmental status among treatments at the sampling endpoint. Moreover, vPS- and ePS-fed larvae showed comparable growth, whereas the endocrine-related transcriptional response was markedly stronger in the ePS group. These observations suggest that developmental-stage variability alone is unlikely to account for the treatment-dependent transcriptional pattern observed.

Regarding neurophysiology, ingestion of polystyrene resulted in a significant inhibition of AChE enzymatic activity, most pronounced in the ePS group. Notably, neither AChE nor AChR transcripts differed between treatments, indicating that the inhibition is likely functional rather than transcriptionally mediated. Such a pattern is consistent with results reported for dragonfly larvae exposed to PS nanoplastics, where AChE activity was strongly suppressed despite no evidence of changes in cholinergic gene expression [29]. In these organisms, reduced AChE activity was interpreted as indicative of neurotoxicity with potential implications for neuromuscular control. Since AChE is essential for cholinergic neurotransmission through the hydrolysis of acetylcholine [30], its impairment can compromise multiple physiological functions in insects, including tenebrionidae. Interestingly, similar reductions in AChE activity have been reported in *Eisenia fetida* exposed to organophosphate and carbamate pesticides, with dose- and time-dependent inhibition accompanied by morphological and behavioral alterations [31]. Overall, AChE inhibition, well-documented in soil and terrestrial invertebrates exposed to cholinesterase-inhibiting pesticides [32,33], provides a reliable indicator of neurochemical disruption and environmental contamination. As AChE activity was assessed in whole-body homogenates, the measured response represents an organism-level evaluation and may partially underestimate alterations occurring specifically in neural tissues, where the enzyme is particularly abundant. Nevertheless, the marked reduction in AChE activity, especially in ePS-exposed larvae, indicates a biologically relevant impairment of cholinergic function. Taken together, the combined endocrine and neurochemical effects observed here point to a dual mode of action of ePS: disruption of ecdysteroid/JH signaling, leading to impaired developmental progression; suppression of AChE activity, suggestive of cholinergic neurotoxicity. The more pronounced biological responses observed in larvae exposed to ePS relative to vPS indicate that field-recovered (environmental) PS represents a biologically more complex exposure matrix than virgin material. This difference may reflect the combined influence of environmentally acquired contaminants and other modifications accumulated during environmental exposure, rather than the pristine polymer alone. Similar weathering-dependent toxicity has been highlighted in previous studies demonstrating that particle aging modifies both physicochemical properties and biological interactions. In contrast, the effect on cholinergic transmission, also observed in vPS, clearly indicates a pesticide driven mechanism given the HCCPD levels found in these particles.

Overall, the results indicate that field-collected polystyrene can interfere with critical physiological pathways involved in growth, metamorphosis, and neuromuscular function. It should also be acknowledged that differences in nutrient availability between the conventional and PS-based diets may have contributed to the reduced growth observed in PS-fed larvae, although this factor alone is unlikely to account for the overall pattern of molecular and biochemical alterations. These outcomes align with the growing body of literature showing that micro- and nanoplastics act as biologically active pollutants capable of inducing oxidative stress, endocrine disruption, and neurotoxicity in aquatic invertebrates. The dual mode of action observed for ePS—i.e., disruption of ecdysteroid/JH signaling and inhibition of AChE activity—raises potential concerns about the health status of larvae employed in plastic waste biodegradation processes. Interference with ecdysteroid and juvenile hormone pathways is likely to impair normal developmental progression, potentially leading to delayed molting, incomplete metamorphosis, growth inhibition and altered life-history trajectories. In parallel, the suppression of AChE activity is indicative of cholinergic neurotoxicity, an effect that is particularly alarming given the key role of AChE in regulating locomotion, feeding, sensory integration and behavior. Evidence from different insect models shows that anticholinesterase effects can result in high mortality, growth impairment, malformations, reduced fertility and behavioral disorders.

## 4. Materials and Methods

### 4.1. Experimental Design and Larval Exposure

*Zophobas morio* larvae (average initial body weight 770 ± 30 mg) were obtained from a commercial breeder and acclimated under controlled environmental conditions (26.8 ± 0.5 °C; 65 ± 5% relative humidity; and 12:12 h light/dark cycle) before the experiment. The experimental protocol was largely consistent with that previously validated by Cocci et al. [2], with slight modifications to the rearing conditions, including reduced humidity and stocking density, aimed at minimizing the high mortality rates previously observed under the original experimental conditions. A total of 210 larvae were randomly allocated to three dietary treatments (*n* = 70 larvae per treatment) in 60 × 60 × 30 cm Vision Cages. Each cage housed two boxes (BraPlast; 3L; Herp Italia, Verona, Italy), each containing 35 larvae, and was assigned to one of the following experimental groups: control, field-recovered (environmental) polystyrene (ePS), and clean PS (vPS). Larvae were fed ad libitum for 28 days with: (1) bran and oatmeal supplemented with fresh carrot (control); (2) virgin, clean polystyrene supplemented with carrot (vPS); or (3) field-recovered (environmental) polystyrene supplemented with carrot (ePS), as reported in Cocci et al. [2]. Fourier-transform infrared spectroscopy was previously used for rapid identification and characterization of the PS [2]. The clean PS was recovered from uncontaminated newly synthesized PS seed trays. The ePS was collected from a coastal site known to be impacted by agricultural runoff. Samples used for chemical, molecular and biochemical analyses were collected from both rearing boxes to ensure balanced representation of each treatment. Larval developmental status was monitored by morphological examination during the feeding trial and was further assessed at the sampling endpoint according to previously described criteria for Zophobas and related tenebrionid larvae [34,35]. No evident treatment-related differences in larval developmental stages were observed at the end of the exposure period. Larvae were then sampled and immediately frozen at −80 °C for molecular and biochemical analyses.

### 4.2. Pesticides Analysis

Sample preparation was based on the QuEChERS method [36,37,38,39]. Briefly, polystyrene feed (0.5 g) and whole-body larvae (without exoskeleton) were weighed and homogenized into a glass centrifuge tube, and acetonitrile was added to the tube. Then, the mixture was vortexed for 1 min and MgSO_4_ and C_2_H_3_NaO_2_ were added to the tube. The sample was additionally vortexed for 1 min and centrifuged at 4500 rpm for 5 min at 10 °C. Samples were then frozen at −20 °C to remove fatty acids [40]. The supernatants were transferred into a glass tube and evaporated to near dry. After that, the extracts were dissolved with 1 mL of the mobile phase solvent. The EPA 505/525 updated pesticide mix B (hexachlorocyclopentadiene, hexachlorobenzene, atrazine, heptachlor, heptachlor epoxide isomer B, γ-chlordane, α-chlordane, endrin, and methoxychlor) and the organophosphorus pesticide mix (dichlorvos, mocap, disulfoton, methyl parathion, ronnel, dursban, tokuthion, and guthion) standards were supplied by Merck (Supelco^®^, Merck, Darmstadt, Germany; Product ID: 47728-U, 48391). Stock solutions of pesticides were prepared by dissolving pure standard compounds in HPLC-grade methanol. The samples were filtered through 0.2 μm syringe filters (Phenomenex Inc., Torrance, CA, USA; Phenex™). HPLC analysis was performed using an HPLC system (Variant ProStar 240 equipped with a Varian ProStar 310 UV-VIS; Bio-Rad Laboratories, Hercules, CA, USA) with appropriate reference standards (Table 1). An isocratic elution was performed to optimize the chromatographic separation of the pesticides. A mixture of water (20%) and acetonitrile (80%) was used as the mobile phase. The total run time for EPA 505/525 updated pesticide mix B and for organophosphorus pesticide mix were 20 min and 40 min, respectively, with an optimal flow rate of 0.6 mL/min. Separation was achieved on a C18 column (5 μm, 250 mm × 4.6 mm; Luna^®^, Phenomenex Inc., Torrance, CA, USA). Sample aliquots of 20 μL were injected onto the HPLC column. The absorption wavelength chosen was 220 nm. The efficiency and the accuracy of the analytical procedures were tested using different reference materials and the recovery fell within the confidence interval (95%). The LODs and the LOQs were calculated according to ICH Q2B.

### 4.3. Molecular Analyses

Total RNA was extracted from whole-body larvae using the QIAzol Lysis Reagent (Qiagen, Hilden, Germania) according to the manufacturer’s specifications. The complementary DNA (cDNA) was synthesized from 2 μg of total RNA using the All-In-One 5X RT MasterMix with gDNA Removal (abm^®^, Applied Biological Materials Inc., Richmond, BC, Canada). The reverse transcription reaction was set up by adding 4 μL of 5X All-In-One RT MasterMix and nuclease-free H_2_O. Samples were incubated at 37 °C for 15 min and then at 60 °C for 15 min. A SYBR Green Real-time Fluorescent Quantitative PCR system (BFQP-4850, Bigfish Bio-tech Co, Hangzhou, Cina) was performed for the molecular analyses with primers for ecdysteroid signaling pathway (EcR, USP, E74, and E75) and juvenile hormone-responsive (Met and Kr-h1) target genes (Table 2). Glyceraldehyde-3-phosphate dehydrogenase (GAPDH) was selected as a reference gene [41]. Primer sequences for EcR, E75, Met, Kr-h1 and GAPDH were adopted from Toga et al. [41], who characterized these endocrine-related genes in *Zophobas atratus*. Primers for E74, USP, AChE and AChR were designed *de novo* using the NCBI Primer-BLAST tool (NIH, version 2.5.0) against the corresponding available *Tenebrio molitor* sequences (GenBank accession numbers: XM_069054586; AJ251542; and XM_069053283) and *Zophobas morio* sequence (GenBank accession number: XM_064076781). The reaction mixture contained 10 μL of BlasTaq™ 2X qPCR MasterMix (abm^®^), 0.5 μL of both 10 μM primers (sense/antisense), 2 μL of cDNA template and nuclease-free H_2_O at an amount of up to 20 μL. Thermal cycling consisted of 3 min at 95 °C, followed by 40 cycles of 15 s at 95 °C and 60 s at 60 °C. The specificity and sensitivity of the primer pairs was confirmed by applying the melting curve analysis produced by the integrated software and verified with agarose gel electrophoresis.

### 4.4. Acetylcholinesterase (AChE) Activity Evaluation

Acetylcholinesterase hydrolysis acetylcholine was used to produce choline and acetic acid. Then, choline reacts with sulfydryl substrate to form TNB (Sym-Trinitrobenzene), a yellow compound which can be measured at 412 nm. The AChE activity was calculated indirectly using the Acetylcholinesterase Activity Assay Kit (MyBioSource, Inc.; San Diego, CA, USA; MBS2540435), according to the manufacturer’s protocol.

### 4.5. Statistical Analysis

All statistical analyses were performed using GraphPad Prism 6.0 (GraphPad Software, Inc., San Diego, CA, USA). Individual measurements obtained from both replicate rearing boxes were retained and jointly summarized for descriptive and graphical purposes. Consistency of the responses between replicate boxes within each treatment was assessed by inspection of box-specific distributions and mean values, with no statistically detectable box-dependent differences observed. Treatment comparisons were based on box-level summary values. Differences among treatments were assessed by one-way ANOVA followed by Dunnett’s multiple-comparison test versus the control group. Given the limited number of independent experimental units, inferential outcomes were interpreted cautiously and together with the consistency and magnitude of the observed responses. Values of *p* < 0.05 were considered statistically significant.

## 5. Conclusions

The present study suggests that ePS can transfer associated contaminants to *Z. morio* larvae during ingestion and is associated with coordinated alterations in transcriptional markers of developmental endocrine signaling and AChE activity. These responses were more pronounced than those observed with virgin PS and indicate a broader toxicological burden associated with field-recovered PS. Although the present design does not resolve the relative contribution of individual contaminants and the weathering process of plastic waste, the results identify endocrine-related signaling and cholinergic function as sensitive endpoints of ePS exposure. Consequently, any strategy that relies on insect larvae for plastic waste management should carefully consider the sublethal and cumulative impacts of plastic-associated toxicity and should integrate ecotoxicological endpoints into the evaluation of their environmental and biotechnological applicability.

## Figures and Tables

**Table 1:** Concentrations (µg g^−1^, mean ± SD) of organochlorine and organophosphorus pesticides detected in field-recovered (environmental) polystyrene (ePS), virgin polystyrene (vPS), and in *Zophobas morio* larvae after dietary exposure. Values below the limit of quantification are indicated as <loq.

**EPA 505/525 Updated Pesticide Mix B**	**HCCPD**	**Atrazine**	**γ-Chlordane**	**Methoxychlor**
ePS	8.65 ± 1.25	1.85 ± 0.51	2.65 ± 0.8	2.55 ± 0.61
vPS	2.55 ± 0.65	<loq	<loq	<loq
Larvae ePS	1.28 ± 0.55	0.32 ± 0.28	0.71 ± 0.55	1.03 ± 0.63
Larvae vPS	0.19 ± 0.08	<loq	<loq	<loq
**Organophosphorus Pesticide Mix**	**Mocap**	**Ronnel**		
ePS	4.26 ± 0.72	3.69 ± 0.97		
vPS	<loq	<loq		
Larvae ePS	1.09 ± 0.56	0.78 ± 0.47		
Larvae vPS	<loq	<loq		

HCCPD: hexachlorocyclopentadiene. Additional target analytes screened but not detected (n.d.): hexachlorobenzene, heptachlor, heptachlor epoxide isomer B, α-chlordane, endrin, dichlorvos, disulfoton, methyl parathion, Dursban, Tokuthion and Guthion.

**Table 2:** Primer sequences (5′–3′) used for quantitative real-time PCR analysis of genes involved in the ecdysteroid signaling pathway (EcR, USP, E74, and E75), juvenile hormone response (Met and Kr-h1), and the reference gene (GAPDH).

Gene	Primer Sequences (5′–3′)	References	Efficiences (%)
EcR	TCTGGGACTTGGACTTGAACG	[41]	97.5
	ATTATGGTGTGTCCGGGTATCC		
USP	GTCTCCTCAGTTTCAGCCCC	PrimerBlast (NIH)	95.8
	CGCAGATCGAGCAGAGATGT		
E74	GACACCCGAAGACTAGTGCC	PrimerBlast (NIH)	96.7
	CACTGCACTGAACACTACCA		
E75	ATCCCGGACCTTGAATTCGAC	[41]	98.1
	AAGAAGCCCTTGCATCCTTC		
AChE	GCCTTTGCTAGATTCGCTGC	PrimerBlast (NIH)	91.8
	GCTCGAGGACCTTTCCCAAT		
AChR	GGAGAAGTGCGCTGTGTAGT	PrimerBlast (NIH)	92.4
	GGAACCAAAACGGACAGTGC		
Met	GCCTTGCGAATTCTACGGTAAC	[41]	95.3
	TGTTACTGGGAGGATGATCTGC		
Kr-h1	ACACATGCGCATACATACCG	[41]	95.8
	TTTGGCACACAGTACAGACG		
GAPDH	TCGTGCTTCTTTGGAACGTG	[41]	96.8
	CAACCAAGAATCCACCTTCTGC		

## Data Availability

The original contributions presented in this study are included in the article. Further inquiries can be directed to the corresponding author.
